# Hepatic lipids promote liver metastasis

**DOI:** 10.1172/jci.insight.136215

**Published:** 2020-09-03

**Authors:** Yongjia Li, Xinming Su, Nidhi Rohatgi, Yan Zhang, Jonathan R. Brestoff, Kooresh I. Shoghi, Yalin Xu, Clay F. Semenkovich, Charles A. Harris, Lindsay L. Peterson, Katherine N. Weilbaecher, Steven L. Teitelbaum, Wei Zou

**Affiliations:** 1 Division of Anatomic and Molecular Pathology, Department of Pathology and Immunology, and; 2 Division of Oncology, Department of Medicine, Washington University School of Medicine, St. Louis, Missouri, USA.; 3 Center for Translational Medicine, The First Affiliated Hospital of Xi’an Jiaotong University, Xi’an, China.; 4 Division of Laboratory and Genomic Medicine, Department of Pathology and Immunology,; 5 Department of Radiology,; 6 Division of Endocrinology, Metabolism & Lipid Research, Department of Medicine, and; 7 Division of Bone and Mineral Diseases, Department of Medicine, Washington University School of Medicine, St. Louis, Missouri, USA.

**Keywords:** Hepatology, Oncology, Cancer, Obesity

## Abstract

Obesity predisposes to cancer and a virtual universality of nonalcoholic fatty liver disease (NAFLD). However, the impact of hepatic steatosis on liver metastasis is enigmatic. We find that while control mice were relatively resistant to hepatic metastasis, those which were lipodystrophic or obese, with NAFLD, had a dramatic increase in breast cancer and melanoma liver metastases. NAFLD promotes liver metastasis by reciprocal activation initiated by tumor-induced triglyceride lipolysis in juxtaposed hepatocytes. The lipolytic products are transferred to cancer cells via fatty acid transporter protein 1, where they are metabolized by mitochondrial oxidation to promote tumor growth. The histology of human liver metastasis indicated the same occurs in humans. Furthermore, comparison of isolates of normal and fatty liver established that steatotic lipids had enhanced tumor-stimulating capacity. Normalization of glucose metabolism by metformin did not reduce steatosis-induced metastasis, establishing the process is not mediated by the metabolic syndrome. Alternatively, eradication of NAFLD in lipodystrophic mice by adipose tissue transplantation reduced breast cancer metastasis to that of control mice, indicating the steatosis-induced predisposition is reversible.

## Abstract 2

Nonalcoholic fatty liver disease promotes liver metastasis in mice, likely due to lipid transfer to tumor cells.

## Introduction

Treatment of early-stage cancer, including surgery, hormone therapy, and chemotherapy, has improved substantially, but preventing metastasis remains a more elusive target. Breast cancer, the most common malignancy in women, recurs in 1 of 5 patients, despite ideal therapy. Luminal breast cancer, the most common type of breast cancer, preferentially metastasizes to bone while lungs and liver represent the second and third most common sites (1, 2). Understanding the microenvironment of these tissues is likely to provide insights into their predisposition for cancer seeding.

Obesity is expected to affect 1 in 2 adults by 2030 (3) and is associated with increased incidence of at least 13 types of cancer (4) and poorer prognosis (5); yet the mechanisms of these associations remain unresolved. There is, however, experimental evidence that the products of adipocytes, juxtaposed to the tumor, promote its growth. The means by which obese adipocytes enhance tumor growth is postulated to involve inflammatory factors, fatty acid transfer, and/or direct interaction with the tumor (6).

The skeleton is the primary target of metastatic breast cancer, but the relationship between bone mass and metastatic predisposition is unknown. To address this issue, we turned to DTA-Stop-flox-Adipoq Cre “fat-free” (FF) mice, which lack brown adipose tissue (BAT) and white adipose tissue (WAT) via germline depletion (7) and are substantially osteosclerotic. However, we found no effect of enhanced bone mass on breast cancer metastasis to the skeleton. The mice in which we tested this hypothesis have severe hepatic steatosis, and surprisingly, it and a variety of other models of nonalcoholic fatty liver disease (NAFLD) have a marked predisposition to liver metastasis, which is unusual in nonsteatotic mice. Thus, pathological lipid accumulation has the capacity to profoundly increase liver metastasis. Furthermore, it appears that in contrast to previous reports implicating inflammation (8), uncomplicated steatosis promotes liver metastasis by providing the products of lipolysis as a source of energy, which is used by tumor cells mainly via mitochondrial oxidation. Importantly, the histology of human liver metastasis suggests the same may occur in humans. Thus, a reciprocal activating relationship exists between steatotic hepatocytes and metastatic cancer. In this circumstance, tumor induces lipolysis of hepatic triglycerides, which are transferred to cancer cells and ultimately serve as an energy source for tumor growth. Given steatosis is preventable and we find its eradication eliminates metastatic predisposition, if our observations translate to patients, the implications would be significant.

## Results

### Steatotic mice are predisposed to hepatic metastasis.

**Figure 1:**
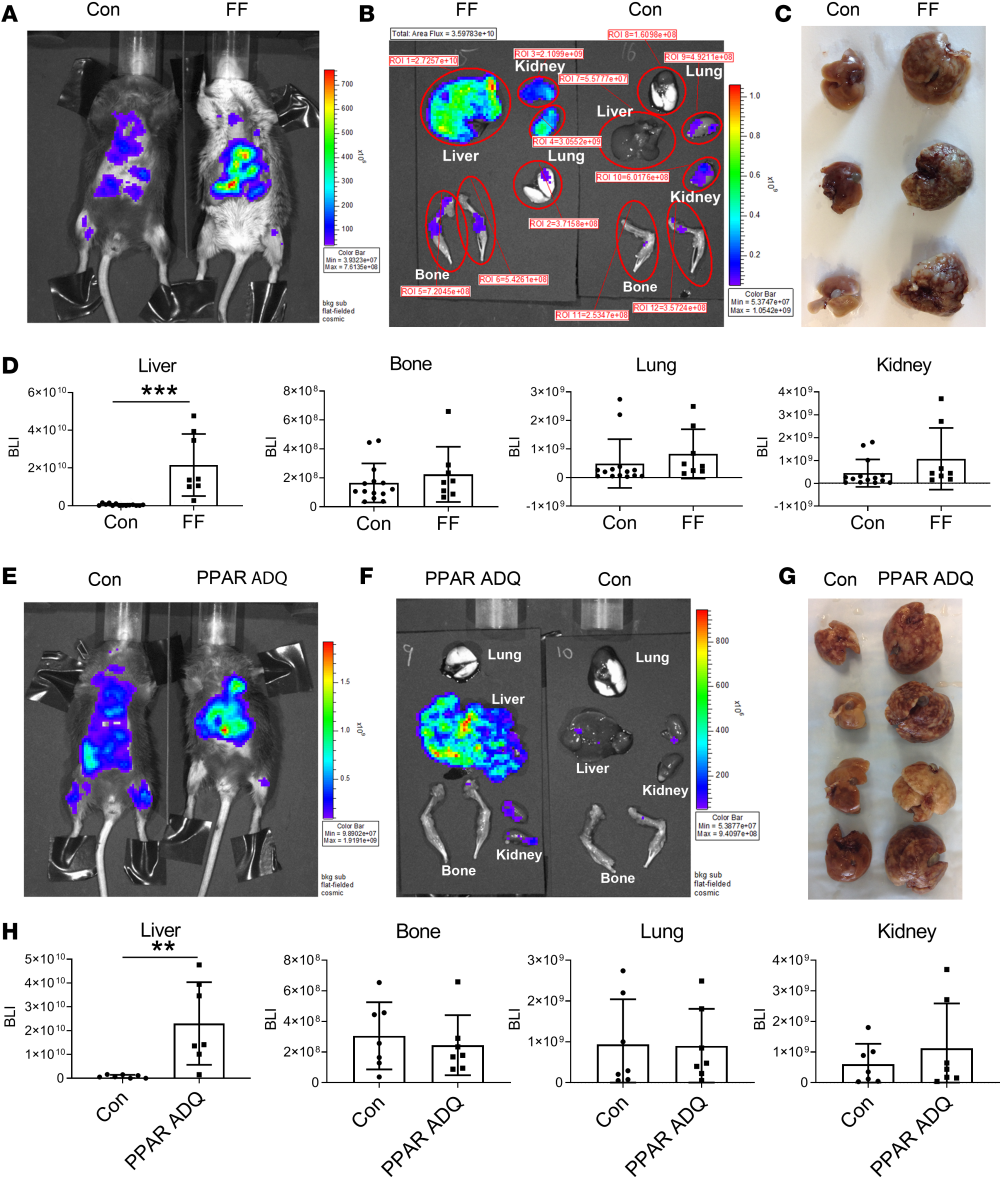
Lipodystrophic mice are predisposed to hepatic metastasis. (**A**–**D**) Two-month-old FF and Con mice were injected with Bo1 cells via left ventricular chamber, and 12 days later tumor burden was analyzed. (**A**) In vivo BLI image; (**B**) ex vivo image of liver, bone, lung, and kidney; (**C**) gross appearance of Con and FF liver; and (**D**) quantification of tumor burden in liver, bone, lung, and kidney of Con and FF mice. *n* = 8–13. ROI, region of interest. (**E**–**H**) Two-month-old PPAR ADQ and Con mice were injected with Bo1 cells via left ventricular chamber, and 12 days later tumor burden was analyzed. (**E**) In vivo BLI image; (**F**) ex vivo image of liver, bone, lung, and kidney; (**G**) gross appearance of Con and FF liver 12 days after tumor injection; and (**H**) quantification of tumor burden in liver, bone, lung, and kidney of Con and FF mice. *n* = 7. Data are presented as mean ± SD. **P < 0.01, ***P < 0.001 as determined by unpaired 2-tailed *t* test.

Given the substantial osteosclerosis in FF mice, they provided an opportunity for determining the effects of increased bone mass on skeletal metastasis. We therefore administered osteolytic PyMT-Bo1-GFP-Luc (Bo1) breast adenocarcinoma cells, via intracardiac injection, to FF mice, which exhibit a virtually complete depletion of WAT and BAT, as well as to their Cre littermates (Con) (7). Contrary to our hypothesis, 12 days after injection, skeletal metastases, determined by in vivo and ex vivo bioluminescence imaging (BLI) and histology, were indistinguishable in fat-depleted and control mice (Figure 1, A, B, and D; and Supplemental Figure 1; supplemental material available online with this article; https://doi.org/10.1172/jci.insight.136215DS1). The same held true regarding lung and kidney tumor abundance. While BLI and gross and microscopic examination revealed a paucity of hepatic metastasis in control mice, breast cancer was abundant in the livers of FF mice (Figure 1, A–D). To confirm the association of lipodystrophy and hepatic metastasis, we administered Bo1 cancer cells to mice conditionally deleted of PPARγ, which is essential for adipocyte differentiation in all fat depots, using adiponectin-Cre (PPAR ADQ). In fact, the liver-predominant metastatic pattern of both lipodystrophic models was indistinguishable (Figure 1).

**Figure 2:**
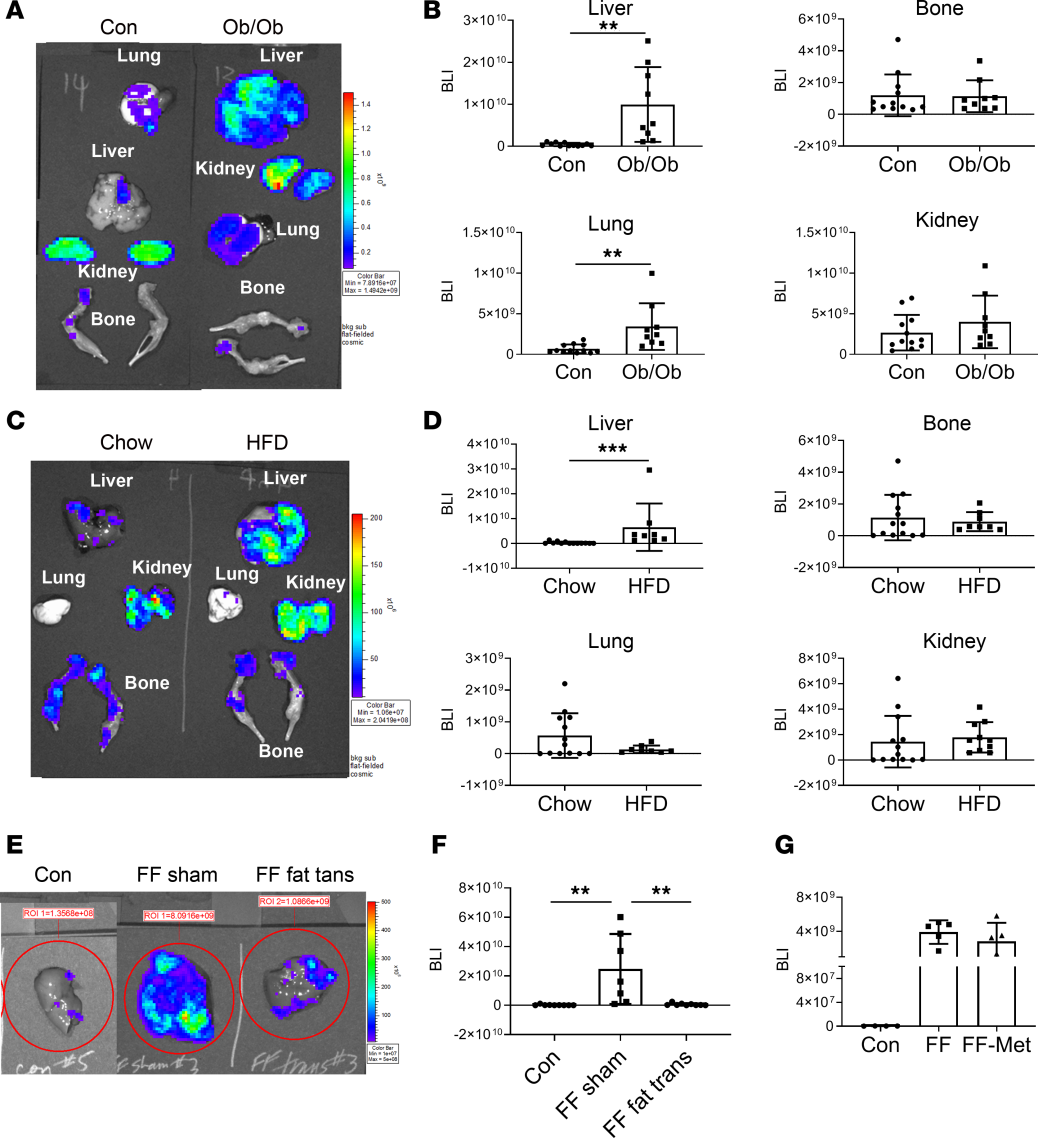
Obese mice are predisposed to hepatic metastasis. (**A** and **B**) Two-month-old leptin^–/–^ (*ob/ob*) and control mice were injected with Bo1 cells via left ventricular chamber, and 12 days later, tumor burden was analyzed. (**A**) Ex vivo image of liver, bone, lung, and kidney. (**B**) Quantification of tumor burden in liver, bone, lung, and kidney. *n* = 9–11. (**C** and **D**) WT mice were fed chow or HFD for 3 months, after which Bo1 cells were injected intracardiacally. (**C**) Ex vivo image of liver, bone, lung, and kidney and (**D**) quantification of tumor burden in liver, bone, lung, and kidney 12 days later. *n* = 8–13. (**E** and **F**) FF mice were injected intracardiacally with Bo1 cells 6 weeks after fat transplantation. (**E**) In vivo BLI image and (**F**) quantification of tumor burden in liver 12 days later. *n* = 8–9. (**G**) Following 2 months of metformin feeding, FF mice were injected with Bo1 cells. BLI quantification of liver tumor burden 12 days after injection. *n* = 4–5. Data are presented as mean ± SD. **P < 0.01, ***P < 0.001 as determined by unpaired 2-tailed *t* test (**B** and **D**) or 1-way ANOVA test with analysis of variance with Holm-Šidák multiple-comparisons test (**F** and **G**).

Circulating leptin and adiponectin are virtually undetectable in FF mice (7). Because both adipokines are reported to suppress metastasis (9, 10), their absence presented as possible mediators of lipodystrophic predisposition to hepatic tumor. To address this issue, we delivered Bo1 cancer cells to leptin^–/–^ (*ob/ob*) or adiponectin^–/–^ mice. While the absence of adiponectin had no effect on hepatic tumor abundance (Supplemental Figure 2), liver metastasis of leptin-deficient mice mirrored FF, although they also had an increased abundance of lung tumors (Figure 2, A and B). To further explore the possible role of leptin deficiency in promoting steatosis-enhanced metastasis, we administered breast cancer cells to mice fed a high-fat diet (HFD) for 12 weeks, producing obesity. In fact, diet-induced obesity, wherein circulating leptin is increased, yielded the same predisposition to hepatic metastasis as deficiency of the adipokine in *ob/ob* mice (Figure 2, C and D). Combined with the fact that Bo1 cells did not express the leptin receptor but bore the adiponectin receptor, it appears that deficiency of these adipokines, in FF mice, does not mediate liver metastasis.

In keeping with their lipodystrophic state, FF and PPAR ADQ (11) mice are steatotic, raising the possibility that NAFLD promotes metastasis. The fact that all models with enhanced metastasis have severe hepatic steatosis while adiponectin^–/–^ mice, which have normal livers, mirror control supports this hypothesis. This distinction raised the possibility that liver lipids contribute to the organ’s metastatic predisposition and that reversal of steatosis in FF mice would reduce tumor abundance. To this end, we transplanted WAT into FF mice, which eradicates fatty liver disease (7) and eliminates enhanced susceptibility to hepatic breast cancer metastasis (Figure 2, E and F). WAT transplantation also normalizes the metabolic syndrome in FF mice (7), raising the possibility that elimination of hyperglycemia and hyperinsulinemia, both implicated in cancer growth, mediates the reversal of metastatic predisposition (12–14). To determine if metabolic abnormalities likely contribute to steatotic liver metastasis, we fed FF mice metformin, standard therapy for lipodystrophy-associated diabetes. While the drug normalizes glucose tolerance and significantly reduces circulating insulin (7), it did not decrease tumor liver metastasis of FF mice (Figure 2G). Thus, increased tumor metastasis in steatotic livers likely does not reflect mice’s metabolic dysfunction. Finally, intracardiac injection of a second tumor model, B16 melanoma cells, generated markedly increased hepatic tumor nodules similar to breast cancer (Supplemental Figure 3). Thus, the predisposition to steatotic liver metastasis is not restricted to breast cancer.

### Transfer of steatotic lipids to tumor.

**Figure 3:**
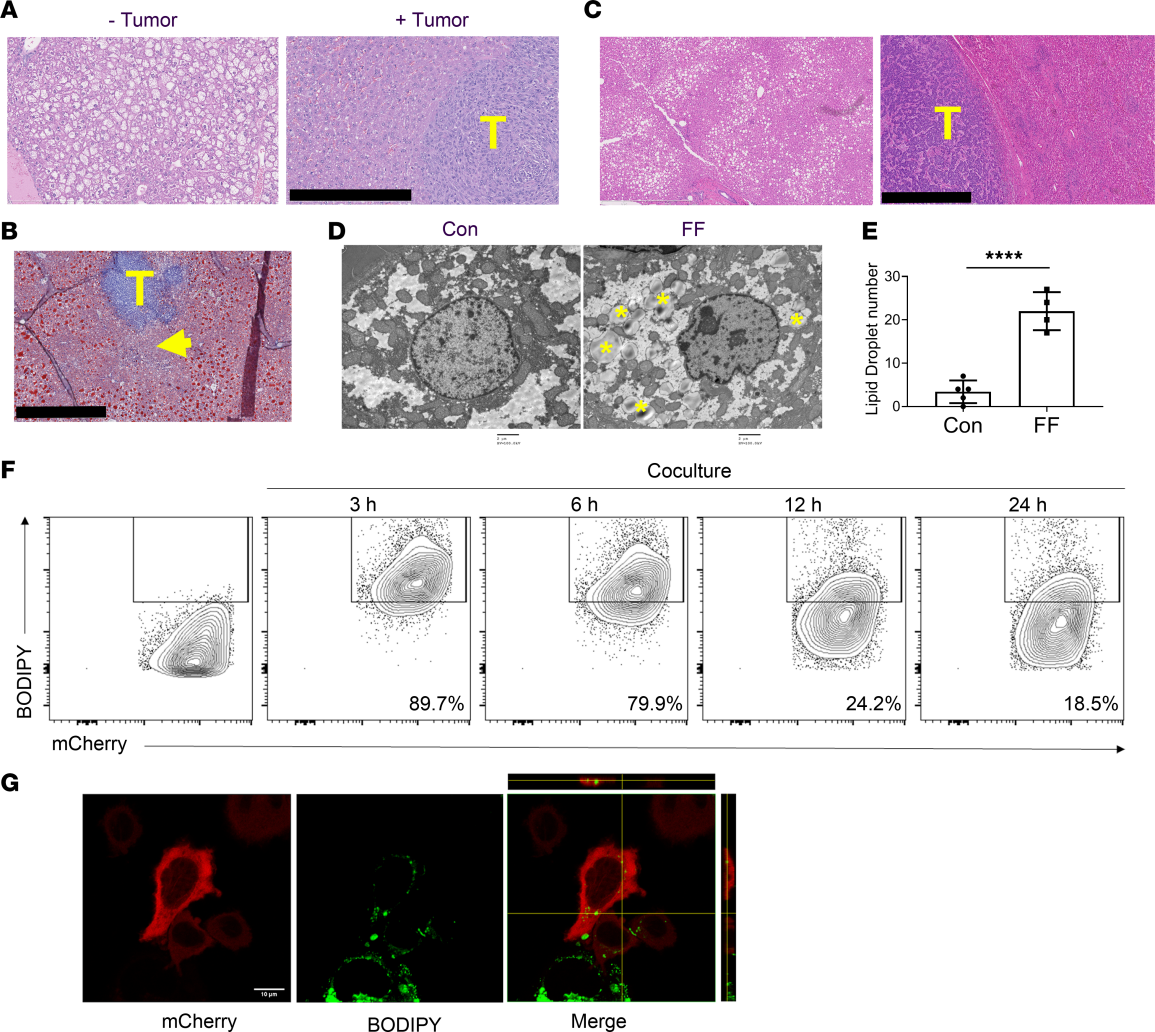
Steatotic hepatocytes transfer lipids to tumor. (**A**) H&E staining of FF steatotic liver before (- tumor) and 12 days after Bo1 intracardiac injection (+ tumor). Note the disappearance of steatosis juxtaposed to tumor. Scale bar: 400 μm. T, tumor. (**B**) Oil red O staining of FF liver 8 days after intracardiac injection of Bo1 cells. Arrow indicates disappearance of lipid staining in hepatocytes juxtaposed to tumor. Scale bar: 1 mm. (**C**) H&E staining of liver of an NAFLD patient with breast cancer metastasis demonstrating areas distal (left panel) and proximal (right panel) to tumor. Scale bar: 1 mm. (**D**) Ultrastructural image of Bo1 cells in the liver of control or FF mice 10 days after intracardiac injection. Yellow asterisks indicate lipid droplets. Scale bar: 2 μm. (**E**) The number of lipid droplets inside a single Bo1 cell in the liver of control and FF mice 10 days after intracardiac injection. *n* = 4–5. (**F**) Flow cytometric analysis of frequency of mCherry-labeled Bo1- cells containing BODIPY derived from HepG2 cells after 0, 3, 6, 12, or 24 hours of coculture. *n* = 3. (**G**) Confocal *Z*-stack imaging of mCherry-labeled Bo1^–^ cell after 3 hours of coculture with BODIPY-treated HepG2 cells. Ten sections of a confocal *Z-*stack were obtained through the height of the cell. The optical section in the middle of cell is presented. The side image on the merged panel is the cross section, and the top image is the longitudinal section of the Bo1^–^ cell present in the middle. The image identifies fluorescence-labeled BODIPY within the tumor cell. Scale bar: 10 μm. Data are presented as mean ± SD. ****P < 0.0001 as determined by unpaired 2-tailed *t* test (**E**).

The predisposition to metastasis of many models of steatosis, including those that are lipodystrophic or obese, raised the possibility that a liver-residing tumor uses hepatic lipids as an energy source. Consistent with transfer from hepatocytes to cancer, the amount of lipid in areas distant from tumor nodules was indistinguishable from non–tumor-injected counterparts while it was markedly reduced in regions juxtaposed to cancer (Figure 3, A and B). Importantly, the same held true in patients (Figure 3C). Conversely, lipid droplets were ultrastructurally more abundant in cancer cells residing in steatotic than in normal liver (Figure 3, D and E). To confirm transfer of lipids from liver to tumor cells, the hepatocyte cell line HepG2 was incubated with the fluorescent fatty acid analog, BODIPY. The HepG2 cells were washed with PBS containing 0.2% fatty acid–free BSA to remove extracellular fatty acids and cocultured with mCherry-labeled Bo1^–^ cells. Tumor cells expressing mCherry and BODIPY were quantitated with time of coculture, by flow cytometry (Figure 3F). After 3 hours and 6 hours of coculture, approximately 90% and 80%, respectively, of Bo1^–^ cells stained with BODIPY. Consistent with metabolism by the tumor cells, BODIPY labeling markedly decreased after 12 and 24 hours of coculture. Confocal microscopy confirmed transfer of BODIPY from HepG2 to Bo1 cells (Figure 3G).

**Figure 4:**
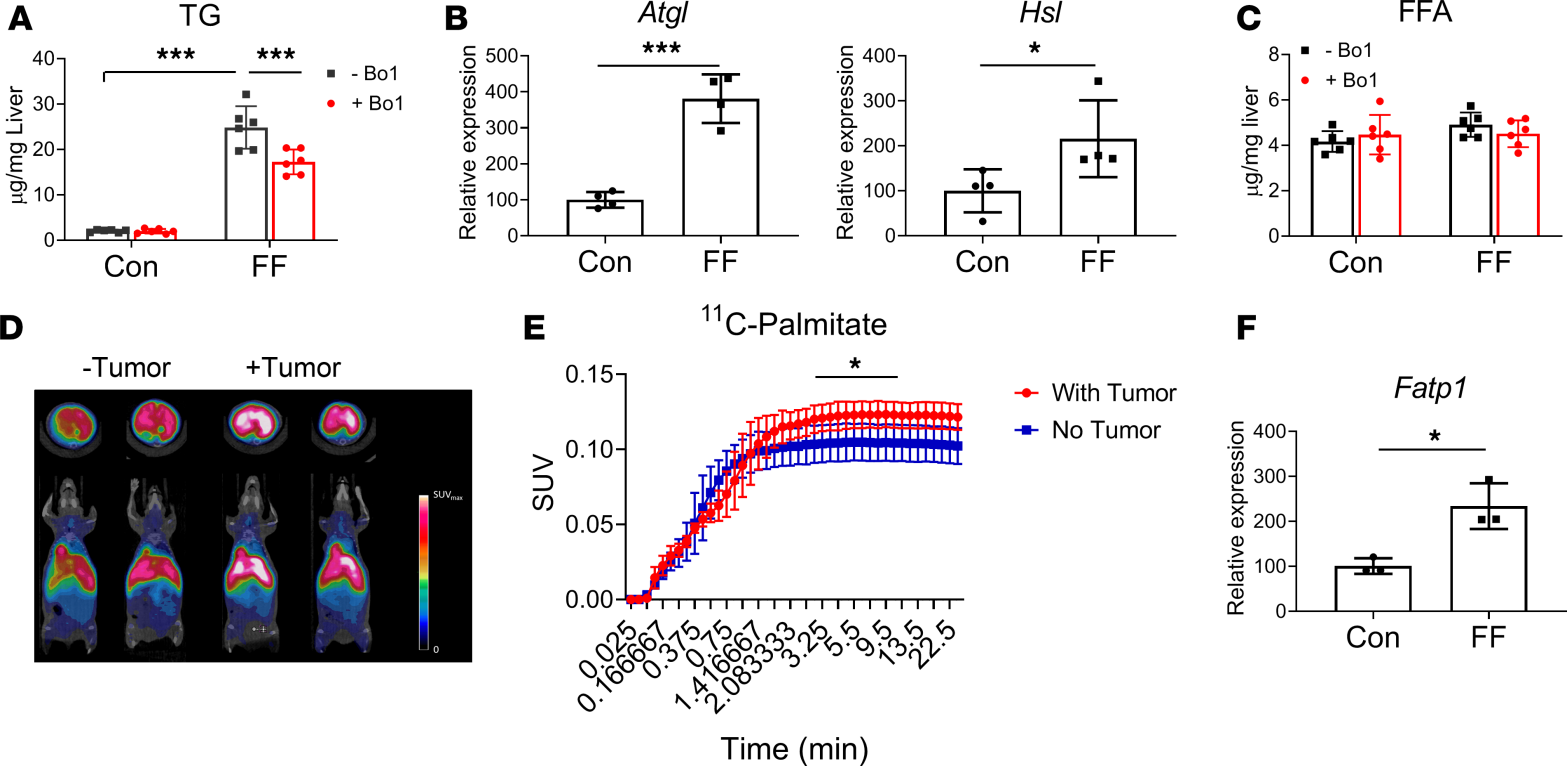
Tumor promotes lipolysis of steatotic liver. (**A**) TG in control or FF liver explants cultured with or without Bo1 cells. *n* = 6. (**B**) Expression of ATGL and HSL in control or FF liver explants cultured with Bo1 cells. *n* = 4. (**C**) FFA in control or FF liver explants cultured with or without Bo1 cells. *n* = 6. (**D**) PET scan of ^11^C-palmitate uptake by control or FF liver 10 days after Bo1 intracardiac injection. (**E**) ^11^C-palmitate uptake quantification. *n* = 4. SUV, standardized uptake value. (**F**) FATP1 mRNA expression in cancer cells exposed to control or steatotic liver–conditioned medium. *n* = 3. Data are presented as mean ± SD. *P < 0.05, ***P < 0.001 as determined by unpaired 2-tailed *t* test (**B**, **E**, and **F**) or 2-way ANOVA test with analysis of variance with Holm-Šidák multiple-comparisons test (**A** and **C**).

As expected, triacylglycerol (TG) was substantially enhanced in livers of FF mice (Figure 4A). Further supporting the concept of transfer of lipids from steatotic liver to cancer cells, TG content of FF but not WT liver, cocultured with Bo1 cells, diminished considerably. Indicating this reduction of TG reflects lipolysis, adipose triglyceride lipase (ATGL) and hormone-sensitive lipase (HSL) gene expression, in the livers of FF mice, was also increased after coculture with Bo1 cells but decreased in the naive condition (Figure 4B and Supplemental Figure 4). Despite this enhanced expression of lipolytic enzymes and decreased TG abundance, free fatty acid (FFA) content in FF liver, cultured with Bo1 cells, was indistinguishable from control (Figure 4C). These observations suggest a scenario in which the products of accelerated lipolysis of TG, in steatotic liver, are transferred to metastatic tumor, where they are metabolized. Consistent with cancer incorporation of the products of lipolysis, ^11^C-palmitate uptake by FF liver was significantly enhanced by the presence of tumor (Figure 4, D and E). To further explore the mechanism whereby steatosis promotes lipid uptake by tumor, we performed quantitative PCR (qPCR) analysis of fatty acid transporter proteins (FATPs) in Bo1 cells exposed to fatty liver–conditioned medium and found only FATP1, which mediates adipocyte to tumor transport, increased (Figure 4F and Supplemental Figure 5) (15). In contrast, CD36, another cancer-activating lipid transporter, is undetectable in Bo1 cells regardless of culture conditions (16).

### Steatosis promotes tumor growth and migration.

**Figure 5:**
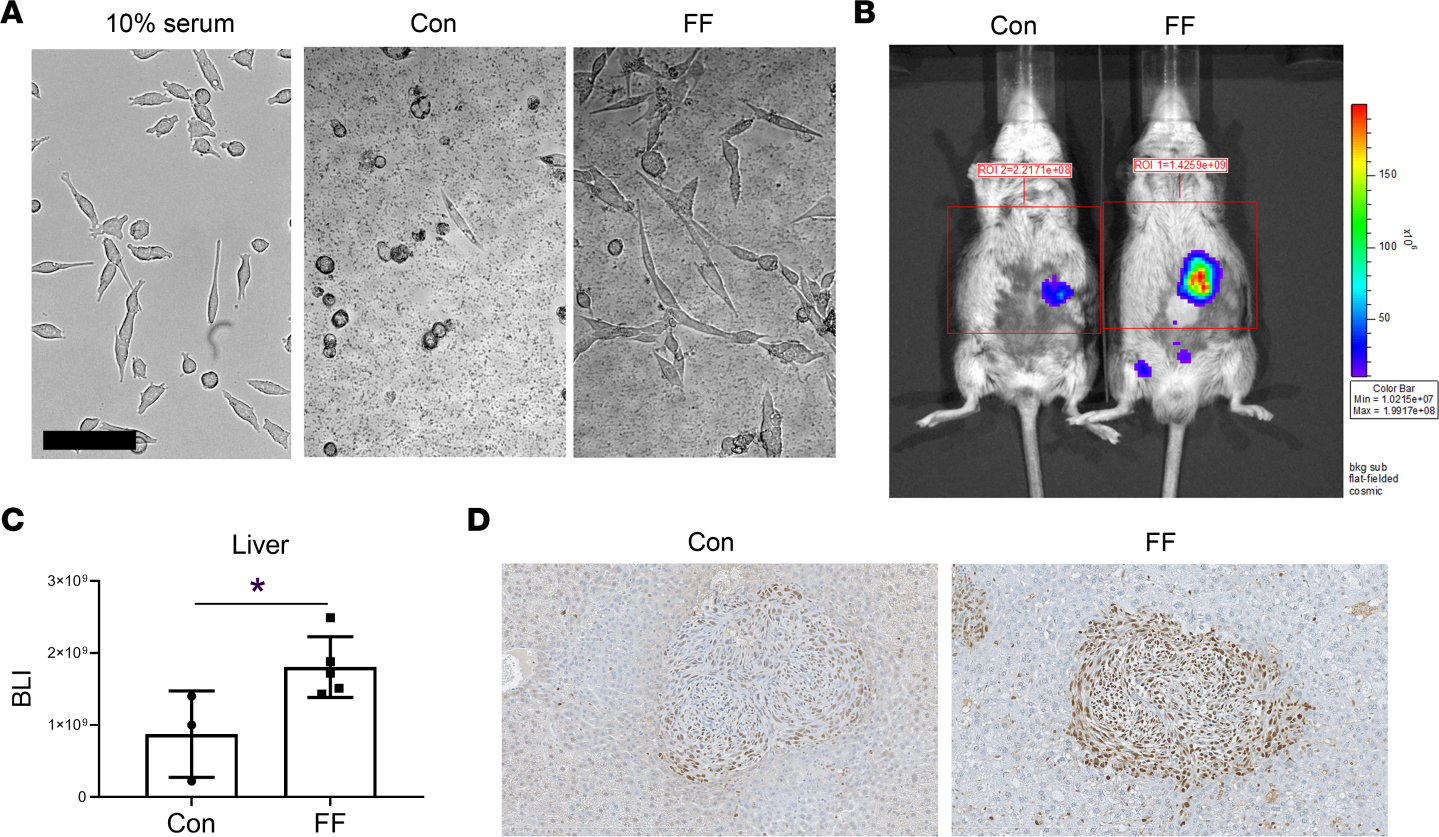
Steatosis promotes tumor growth. (**A**) Morphology of Bo1 cells cultured with Con or steatotic liver of FF mice in serum-free medium; left panel shows the normal Bo1 cells cultured with medium with 10% serum. Scale bar: 100 μm. (**B** and **C**) Bo1 cells were injected directly into livers of 2-month-old FF and control mice. Tumor burden was analyzed 8 days later: (**B**) in vivo BLI image and (**C**) quantification of tumor burden in liver. *n* = 3–5. (**D**) PCNA immunostaining of liver of control or FF mice 12 days after intracardiac injection of Bo1 cells. *n* = 3. Data are presented as mean ± SD. *P < 0.05, as determined by unpaired 2-tailed *t* test (**C**).

Given our evidence of transfer of lipids from steatotic hepatocytes to cancer, we turned to its functional implications. Thus, we asked whether coculture with FF or Con liver has distinct morphological effects on Bo1 cells. Consistent with a product of steatotic hepatocytes directly affecting breast cancer, FF liver normalized the altered shape of tumor cells cultured in serum-depleted medium, while Con liver showed no such effect (Figure 5A).

To assess the impact of steatosis on growth of liver-residing, metastatic breast cancer, we directly injected Bo1 cells into the livers of FF and control mice. There was substantially more tumor in the steatotic than control liver 8 days later (Figure 5, B and C). Confirming that tumor abundance in FF injected liver represented accelerated cancer cell replication, proliferating cell nuclear antigen (PCNA) was increased (Figure 5D).

**Figure 6:**
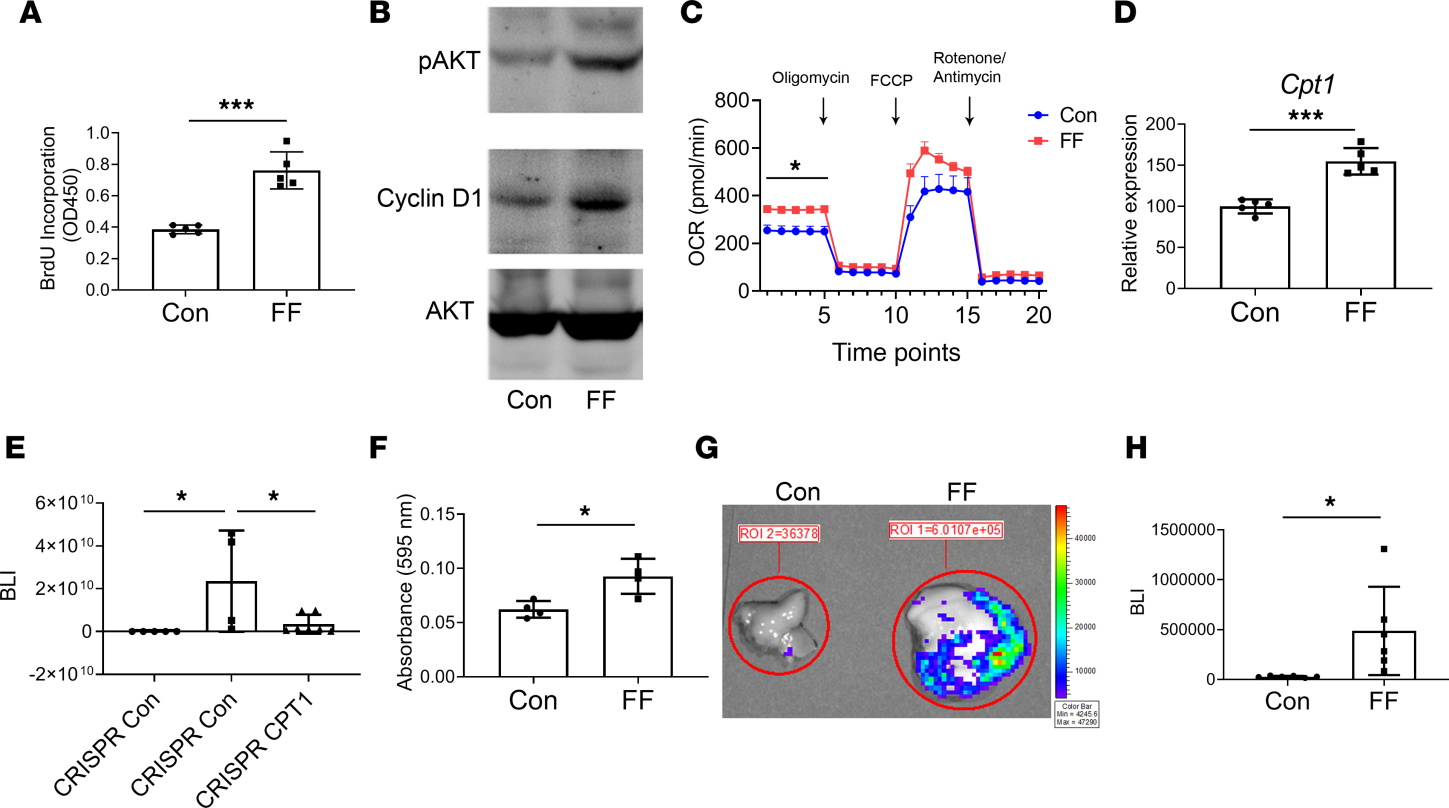
Steatosis promotes metastatic tumor growth. (**A**) BrdU incorporation and (**B**) AKT and cyclin D1 immunoblot of Bo1 cells treated with lipids (100 μg/mL) from control or FF liver. *n* = 5. (**C**) Seahorse oxygen consumption rate (OCR) of Bo1 cells cocultured with control or fatty liver. *n* = 9. (**D**) Carnitine palmitoyltransferase 1 (CPT1) mRNA expression in Bo1 cells cocultured with control or FF liver. *n* = 4–5. (**E**) BLI analysis of FF liver 12 days after intracardiac injection of CRISPR control or CRISPR-CPT1–knockout Bo1 cells. *n* = 4–7. (**F**) Bo1 cells were cultured with control or FF liver explants in Transwell system and migrating cell number was analyzed. *n* = 4. (**G** and **H**) Two-month-old FF and control mice were injected intracardiacally with Bo1 cells. Tumor burden was analyzed by BLI 24 hours later: (**G**) ex vivo image of liver and (**H**) quantification of tumor burden in liver. *n* = 6. Data are presented as mean ± SD. *P < 0.05, ***P < 0.001 as determined by unpaired 2-tailed *t* test (**A**, **C**, **D**, **F**, and **H**) or 1-way (**E**) ANOVA test with analysis of variance with Holm-Šidák multiple-comparisons test.

To determine if steatosis enhances the tumor growth–stimulating capacity of lipids, we added equal amounts lipids, derived from FF and WT livers, to cultures of Bo1 cells. As indicated by BrdU incorporation and supported by immunoblots of the mitosis-associated signaling molecules, protein kinase B (AKT) and cyclin D1, steatosis enhanced the capacity of lipids to promote tumor growth (Figure 6, A and B). Furthermore, coculture with FF liver increased basal mitochondrial respiration of Bo1 cells as indicated by Seahorse oxygen consumption assay (Figure 6C). Consistent with this observation, gene expression of carnitine palmitoyltransferase 1 (CPT1), a rate-limiting event of mitochondrial β-oxidation, which controls mitochondrial uptake of long-chain acyl-CoAs, was upregulated in Bo1 cells cocultured with fatty liver (Figure 6D). Alternatively, *CPT1* gene deletion in Bo1 cells decreased tumor growth, in vivo (Figure 6E). Thus, transfer of lipid products from steatotic hepatocytes appeared to promote metastatic growth. Likely reflecting robust hepatic gluconeogenesis, FF liver also increased the glycolytic activity (extracellular acidification rate; ECAR) of Bo1 cells (Supplemental Figure 6). Suggesting steatosis also predisposes cancer cell hepatic implantation (i.e., seeding), Transwell coculture documented more Bo1 cells migrating to the fatty than to the normal liver (Figure 6F). Supporting this concept in vivo, BLI documented increased tumor abundance in FF liver only 24 hours after intracardiac injection (Figure 6, G and H). In contrast to tumor growth, however, *CPT1* gene deletion had no effect on cancer cell migration to steatotic liver, indicating it is likely not mitochondria based (Supplemental Figure 7).

## Discussion

Obesity is associated with an increased incidence of primary and metastatic breast and other cancers (17). Patients who are obese have poorer overall and breast cancer–specific survival (18). While a number of mechanisms, including insulin resistance and inflammation, are invoked, the means by which obesity promotes tumorigenesis and compromises survival remain enigmatic (19–21).

The skeleton is the most common site of breast cancer metastasis, and we postulated that its marked bone accrual may render the FF mouse resistant to this complication. We found this not to be the case but observed a striking increase in metastasis to livers of these animals and others that are steatotic regardless of whether they are lipodystrophic or obese. Melanoma also abundantly metastasized to steatotic livers, indicating the fatty organ’s neoplastic predisposition goes beyond adenocarcinoma of the breast.

Because of the endemic nature of the metabolic syndrome, NAFLD is estimated to be present in approximately 25% of Americans (22). The prevalence of NAFLD in patients with breast cancer is even higher, in one study reaching 72% of those treated with systemic therapy (23). Given the prevalence of hepatic metastasis in breast cancer and steatosis, we were surprised by the paucity of information regarding the relationship of the 2 conditions in patients. One clinical study suggests steatosis restricts liver metastasis (24), but another claims it is increased (25).

Histological examination of the metastasis-bearing livers of steatotic mice revealed a marked reduction in lipid droplets in hepatocytes abutting the tumor, suggesting their transfer to cancer cells, where they may serve as an energy source for tumor growth. The same histological features are present in metastasis-bearing human steatotic liver. This model is consistent with relocation of lipids from adipocytes to ovarian and breast cancer cells as well as melanomas to promote growth (26). In fact, proliferating cancer cells may preferentially use exogenous fatty acids as their membrane lipid source (27). We therefore hypothesized that steatosis facilitates hepatic metastasis by providing lipid products as an energy source to tumors. Consistent with this conclusion, Seahorse analysis of cancer cells, cultured with steatotic or normal liver, indicates β-oxidation is enhanced. Mitochondrial oxidation is also increased in other forms of cancer (28), particularly when lipids, derived from adipocytes, are transferred to malignant cells (29). The functional significance of enhanced β-oxidation in steatotic metastasis is supported by induced expression of *CPT1* in cancer cells cultured with steatotic liver. This enzyme promotes formation of acyl carnitines by catalyzing transfer of the acyl group of a long-chain fatty acyl-CoA from CoA to l-carnitine. Confirming an essential role of oxidation, deletion of *CPT1* in Bo1 cells virtually eliminated the metastatic predisposition of fatty liver.

The likelihood that a liver-residing tumor induces lipolysis in steatotic hepatocytes is fortified by the decline in TG content in FF liver cultured with Bo1 cells. In addition, while lipolytic enzymes are diminished in naive FF liver, they are increased in the presence of cancer. Additionally, while not establishing the incorporating cell is neoplastic, the fact that metastasis stimulates palmitate uptake suggests that by mechanisms to be discovered, tumor may stimulate lipolysis in steatotic liver, the products of which are incorporated into cancer cells to serve as substrates for β-oxidation. The accumulation of lipid droplets in Bo1 cells, juxtaposed to fatty hepatocytes, indicates that as in transfer of adipocyte lipids to breast cancer, the hepatocyte lipolytic products may be metabolized upon incorporation or temporarily stored as TGs (29). We confirmed transfer of lipids to tumor and their subsequent metabolism by coculture of BODIPY-bearing hepatocytes and Bo1 cells. Consistent with steatotic hepatocytes providing fatty acids to cancer cells, FF liver–conditioned medium selectively enhanced Bo1 expression of FATP1, which promotes metastasis by transporting adipocytic lipids to metastatic melanoma (15).

Net metastasis reflects predisposition of cancer to seed target organs and its subsequent proliferation. A number of parameters, such as PCNA expression, establish that growth of cancer, once seeded in fatty liver, is enhanced. It appears that steatotic liver also facilitates seeding of breast cancer, as illustrated by the abundance of tumor in FF mice and its absence in WT, only 24 hours after direct injection. The fact that *CPT1* deletion in tumor cells virtually eliminates steatotic metastasis while not affecting migration suggests that β-oxidation provides the energy for tumor growth but not seeding.

Previous studies conclude that induction of metastasis in steatotic liver is mediated by inflammation. This conclusion is likely most appropriate in the context of nonalcoholic steatohepatitis as hepatic inflammation tends to be minimal in most patients with typical NAFLD. Additionally, hepatocyte-produced serum amyloid A1 and A2, which promote formation of a premetastatic niche in liver, in the context of fibrosis and inflammation, is decreased in FF mice (30). CXCL12/CXCR4 and CXCL16/CXCR6 chemokine signaling also mediates breast cancer progression (31, 32). Although increased CXCL12 and CXCL16 gene expression were observed in fatty liver, deletion of CXCR6 and CXCR4 in Bo1 cells failed to reduce metastasis (data not shown). Thus, while other factors, such as the low-grade inflammation of obesity, may contribute to the predisposition of murine NAFLD to metastasis, it is likely the predominant mechanism is lipid transfer.

We believe the present report is first to document that the products of pathologically deposited lipids promote metastasis and do so in a primary organ of tumor seeding. Given the similar histological features of lipid distribution in human metastasis-bearing liver, there is a reasonable likelihood that our observations will translate to patients. If so, the clinical implications are significant as this common metabolic disorder is preventable and reversible, and its diminution may reduce liver metastasis.

## Methods

### Mice.

Adipoq-Cre, PPARγ*^fl/fl^*, adiponectin^–/–^, and leptin^–/–^ mice were purchased from The Jackson Laboratory. FF mice were generated by mating homozygous Lox-stop-Lox-ROSA-DTA mice (7) to those expressing adipoq-Cre. Adipocyte-specific PPARγ deletion mice were generated by mating PPARγ*^fl/fl^* mice with adipoq-Cre (PPAR ADQ). Lox-stop-Lox-ROSA-DTA mice and PPARγ*^fl/fl^* mice were used as control mice of FF mice and PPAR ADQ mice, respectively. C57BL/6 and Leptin^+/–^ mice were used as control mice of adiponectin^–/–^ and leptin^–/–^ mice, respectively. Both FF and PPAR ADQ mice could not survive at room temperature after birth because of lack of BAT, so they were housed at thermoneutral condition (30°C) till weaning age (3 weeks old). All other mice were kept at 22°C on a 12-hour light/12-hour dark cycle. Although no sex differences existed in phenotype, female mice were exclusively used. Mice used in experiments were 8–16 weeks old.

For HFD-feeding studies, mice were fed with chow diet until age 8 weeks and thereafter were randomized into groups that were fed either chow diet or HFD (Research Diets Inc., catalog D12492) for 3 months. Metformin was purchased from MP Biomedicals and dissolved in mouse drinking water (2 g/L) (7). Fresh drinking water with metformin was changed daily for 2 months.

### Cell lines and construction of gene deletion cells and mCherry-labeled cells.

Mouse breast cancer cell lines PyMT-Bo1 (Bo1^–^), PyMT-Bo1-GFP-Luc (Bo1), and PyMT-B6 (B6) (estrogen receptor^+^ cells) (33); B16 murine melanoma cell line (ATCC); human hepatocyte cell line (HepG2, ATCC); and platinum-E (Plat-E, ATCC) retroviral packaging cell line were maintained at DMEM with 10% FBS.

CRISPR guides were created using CRISPR Design (http://crispr.mit.edu). The sequence of guide RNA (gRNA) for control and CPT1 deletion was 5′-ACACGCGCTTCCGCGGCCCGTTCAA-3′ and 5′-GCCCAAAAGCAACGGAATCG-3′, respectively. gRNAs were subcloned into the lentiCRISPR V2 system (34), and viruses were then infected into Bo1 cells. Puromycin selection was used to obtain control clones and clones deficient for CPT1. The gene deletion was determined by T7E1 assay with T7 Endonuclease I (New England BioLabs, catalog M0302).

Retroviral plasmid pMX-RFP-mCherry was transfected transiently into Plat-E packaging cells using PolyJet in vitro DNA transfection reagent (SignaGen Laboratories, catalog SL100688). Virus was collected 48 hours after transfection. Bo1 cells were infected with virus for 24 hours in the presence of 4 μg/mL polybrene (MilliporeSigma). Cells were selected in the presence of 2 μg/mL puromycin for 3 days before use.

### Mouse tumor models.

For systemic administration of cancer, the left ventricular chamber of 2-month-old mice was injected with 1 × 10^5^ PyMT-Bo1-GFP-Luc cells or B16 murine melanoma cells in 50 μL PBS as described previously (35). For direct liver injection, livers of 2-month-old mice were surgically exposed. Fifty thousand PyMT-Bo1-GFP-Luc cells in 10 μL PBS were injected directly into the liver using an insulin syringe with 29-gauge needle. BLI was used to quantify the tumor at indicated times after injection.

### Bioluminescence imaging.

For BLI of live animals, as previously described (35), mice were injected intraperitoneally with 150 μg/g d-luciferin (Biosynth Carbosynth) in PBS, anesthetized with 2.5% isoflurane, and imaged with a charge-coupled device camera-based BLI system (IVIS 100; Caliper Life Sciences; exposure time 1–60 seconds, binning 8, field of view 12, f/stop 1, open filter, anterior side). Signal was displayed as photons/s/cm^2^/steradian. ROIs were defined manually around the legs using Living Image (PerkinElmer) and Igor Pro Software (Version 2.50, WaveMetrics).

### Fat transplantation.

Mature fat depots were transplanted as described before (7). Two-month-old FF mice were anesthetized with isoflurane. Donor WAT fat pads from 6- to 8-week-old WT mice were cut into 100–150 mg pieces. The grafts were implanted subcutaneously through small incisions in shaved skin of the back, with 1 piece per incision. Six pieces of fat graft were implanted into each FF mouse. After surgery the mice were housed individually for a week and then 5 mice per cage. Mice were injected with tumor cells 6 weeks after transplantation.

### Immunoblotting.

Cultured cells were washed twice with ice-cold PBS and lysed in RIPA buffer (MilliporeSigma) containing 1× protease inhibitor cocktail (Roche cOmplete). After incubation on ice for 10 minutes, cell lysates were clarified by centrifugation at 21,000*g* at 4°C for 10 minutes. Then, 40 μg of total lysates were subjected to 10% SDS-PAGE and transferred onto PVDF membranes. Filters were blocked in 0.1% casein in PBS for 1 hour and incubated with primary antibodies (phospho-Akt catalog 4056, AKT catalog 9272, cyclin D1 catalog 2922, all from Cell Signaling Technology) at 4°C overnight followed by probing with fluorescence-labeled secondary antibodies (Jackson ImmunoResearch Laboratories). Proteins were detected with the Odyssey Infrared Imaging System (LI-COR Biosciences).

### Immunohistochemical staining.

Paraffin sections (5 μm) from mice were rehydrated and treated with 0.3% hydrogen peroxide in methanol for 15 minutes to suppress endogenous peroxidase activity. Antigen retrieval was achieved by microwaving the sections in 10 mM citrate buffer for 10 minutes followed by gradual cooling to room temperature. Sections were incubated with primary PCNA antibody (PC10, Santa Cruz Biotechnology) overnight at 4°C. Immunostaining was detected using the Histostain-SP Broad Spectrum (DAB) kit (Thermo Fisher Scientific, catalog 95-9743).

### Flow cytometry.

To generate fluorescence-labeled lipids, HepG2 cells were incubated with fluorescent fatty acid analog (C1-BODIPY 500/510 C_12_, Thermo Fisher Scientific, catalog D3823) for 24 hours. Thereafter, HepG2 cells were washed with PBS containing 0.2% fatty acid–free BSA to remove extracellular fatty acids and cocultured with mCherry-labeled Bo1^–^ cells. After 0, 3, 6, 12, and 24 hours’ coculture, the cells were collected and stained with the dead cell exclusion dye ZombieUV (1:600; BioLegend) in PBS. Cells were then incubated with 10 mg/mL FcBlock (clone 24G.2; BD Biosciences) in PBS containing 2.5% heat-inactivated FBS and 2 mM EDTA before being stained for flow cytometric analyses, as previously described (36, 37). Mouse anti–human HLA-A,B,C Antibody (BioLegend; clone W6/32; 1:200) was used to stain HepG2 cells. Flow cytometric analyses were performed with FlowJo (version 10), and cells were gated on singlets and live cells.

### Confocal microscopy.

HepG2 cells were treated with BODIPY 500/510, then cocultured with mCherry-labeled Bo1^–^ cells on microscope cover glass (Fisherbrand, Thermo Fisher Scientific, catalog 12-545-80). After 3 hours of coculture, cells were rinsed with PBS and fixed with 4% formalin. Then the cells were analyzed with a Zeiss LSM880 laser scanning confocal microscope (Carl Zeiss Inc.) equipped with a ×63, 1.4 numerical aperture Zeiss Plan Apochromat oil objective. The argon (excitation 488 nm) and helium neon (excitation 543 nm) lasers were used in obtaining confocal *Z* slices of 0.9 μm through the entire height of cells. ImageJ software (NIH) was used to analyze the images.

### Electron microscopy.

Tumor tissues from Con and FF livers that had been intracardially injected with Bo1 cells for 10 days were cut into about 2 mm size and fixed at 5% glutaraldehyde in 0.16 M collidine buffer overnight. Then samples were washed in sodium cacodylate buffer at room temperature and postfixed in 1% osmium tetroxide (Polysciences Inc.) for 1 hour. Samples were then rinsed extensively in distilled H_2_O (dH_2_O) before en bloc staining with 1% aqueous uranyl acetate (Ted Pella Inc.) for 1 hour. Following several rinses in dH_2_O, samples were dehydrated in a graded series of ethanol and embedded in Eponate 12 resin (Ted Pella Inc.). Sections of 95 nm were cut with a Leica Ultracut UCT ultramicrotome (Leica Microsystems Inc.), stained with uranyl acetate and lead citrate, and viewed on a JEOL 1200 EX transmission electron microscope (JEOL USA Inc.) equipped with an AMT 8-megapixel digital camera and AMT Image Capture Engine V602 software (Advanced Microscopy Techniques).

### Liver lipid extraction.

Liver lipid was extracted following the standard chloroform/methanol/H_2_O protocol (38). Briefly, fresh liver was homogenized on ice, and PBS-methanol (1:1, *v/v*) was added, followed by addition of the same volume of chloroform. The mixture was kept at –20°C for at least 30 minutes. The bottom layer was dried with nitrogen and lipids extracted with toluene-isopropanol (1:3, *v/v*).

### Liver lipid measurement.

Liver explants (50 mg) from Con and FF mice were cocultured with Bo1 cells in Transwell plates. After 24 hours’ coculture, liver explants were collected and sent to the metabolic core, Washington University School of Medicine, for the analysis of triglyceride and FFAs.

### ^11^C-palmitate PET.

Ten days after intracardiac injection of Bo1 cells, FF mice were subjected to PET imaging using cross-calibrated Siemens Inveon PET/CT or Focus F220 scanners. Mice were secured in a custom-designed acrylic restraining device and placed inside the field of view of the scanners. A 30-minute dynamic PET image acquisition was initiated immediately preceding a bolus injection of ^11^C-palmitate via the tail vein. Dynamic images were reconstructed using Ordered Subsets Expectation Maximization algorithm. Images were analyzed by drawing ROIs on livers to ascertain in vivo measures of fatty acid (via ^11^C-palmitate) metabolism. Data derived from ROIs were normalized to standardized uptake values (SUV = activity × [weight of mouse/injected dose]) to account for differences in injected dose and weight of mice.

### RNA isolation and qPCR.

Total RNA from fresh liver or cells was extracted using TRIzol (Invitrogen, Thermo Fisher Scientific) following RNA purification with RNeasy RNA purification kit and RNase free DNase digestion (QIAGEN, catalog 74104). Complementary DNA was synthesized from RNA (1 μg) using the iScript cDNA Reverse Transcription kit (Bio-Rad, catalog 1708890) according to the manufacturer’s instructions. Real-time PCR was performed using the SYBR Green Master Mix kit (Applied Biosystems, Thermo Fisher Scientific, catalog A25741) and the gene-specific primers listed in Table 1. GAPDH was used as a housekeeping gene. PCRs for each sample were performed with 7500 Fast Real-Time PCR System (Applied Biosystems, Thermo Fisher Scientific) using the comparative threshold cycle method for relative quantification.

### Cell mitochondrial stress (Seahorse) assay.

The simultaneous measurements of cellular OCR (in pmol/min) and ECAR (in mpH/min) were performed with a Seahorse Bioscience XF96 Extracellular Flux Analyzer. After Transwell coculture with liver for 24 hours, 50,000 Bo1 cells/well were plated on Seahorse 96-well plates 6 hours before assay. Baseline OCR and ECAR were measured at 5 time points, followed by the sequential injection of oligomycin (final concentration, 1 μM), FCCP (final concentration, 2 μM), and a mixture of rotenone (final concentration, 1 μM) and antimycin A (final concentration, 1 μM).

### Cell migration assay.

Migration assay was performed using 8 μm Transwell filters (Costar, Corning). Briefly, 10 mg of fresh liver tissues were placed in the lower chamber with DMEM and 1 × 10^5^ Bo1 cells in the upper wells; 24 hours later, the cells in the inserts were fixed with 70% ethanol and cells attached to the top surface of the membrane were removed with cotton swabs. Residual cells on the bottom of the insert membrane were stained with 1% crystal violet (Thermo Fisher Scientific). After elusion with 1% SDS, the crystal violet dye was measured by absorbance at 595 nm.

### Statistics.

Data are presented as mean ± SD. Differences between groups were evaluated by unpaired 2-tailed Student’s *t* test or 1-way or 2-way ANOVA test with ANOVA with Holm-Šidák multiple-comparisons test. All experiments were repeated at least 2 times.

### Study approval.

Animal work was performed according to the policies of the Animal Studies Committee at Washington University School of Medicine in St. Louis. Mice were analyzed under approved protocols and were provided appropriate care while undergoing research that complied with the standards in the *Guide for the Use and Care of Laboratory Animals* (National Academies Press, 2011) and the Animal Welfare Act. The unidentified patient biopsy slide did not require IRB approval.

## Author contributions

YL designed and performed experiments and wrote the original draft; XS, NR, YZ, JRB, and YX conducted experiments; KNW designed experiments and reviewed and edited the manuscript; and KIS, CFS, CAH, and LLP reviewed and edited the manuscript. SLT supervised the project and designed experiments and wrote the manuscript; WZ supervised the project and designed and performed experiments and reviewed the manuscript.

## Supplementary Material

Supplemental data

## Figures and Tables

**Table 1:**
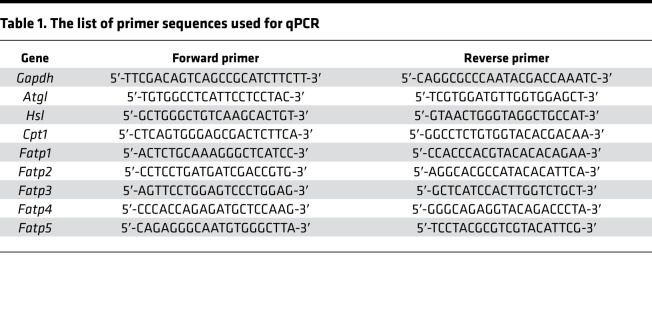
The list of primer sequences used for qPCR

## Version 1 09/03/2020

Electronic publication

## Version 2 09/13/2020

The spelling of Katherine N. Weilbaecher's name was corrected.
