# Supplementary material for: Hepatic lipids promote liver metastasis
Source: JCI Insight. 2020 Sep 3;5(17):e136215. doi: 10.1172/jci.insight.136215 (PMC7487169; doi:10.1172/jci.insight.136215)
Supplement: Supplemental data [file jciinsight-5-136215-s269.pdf]

Supplemental figures:

Figure S1

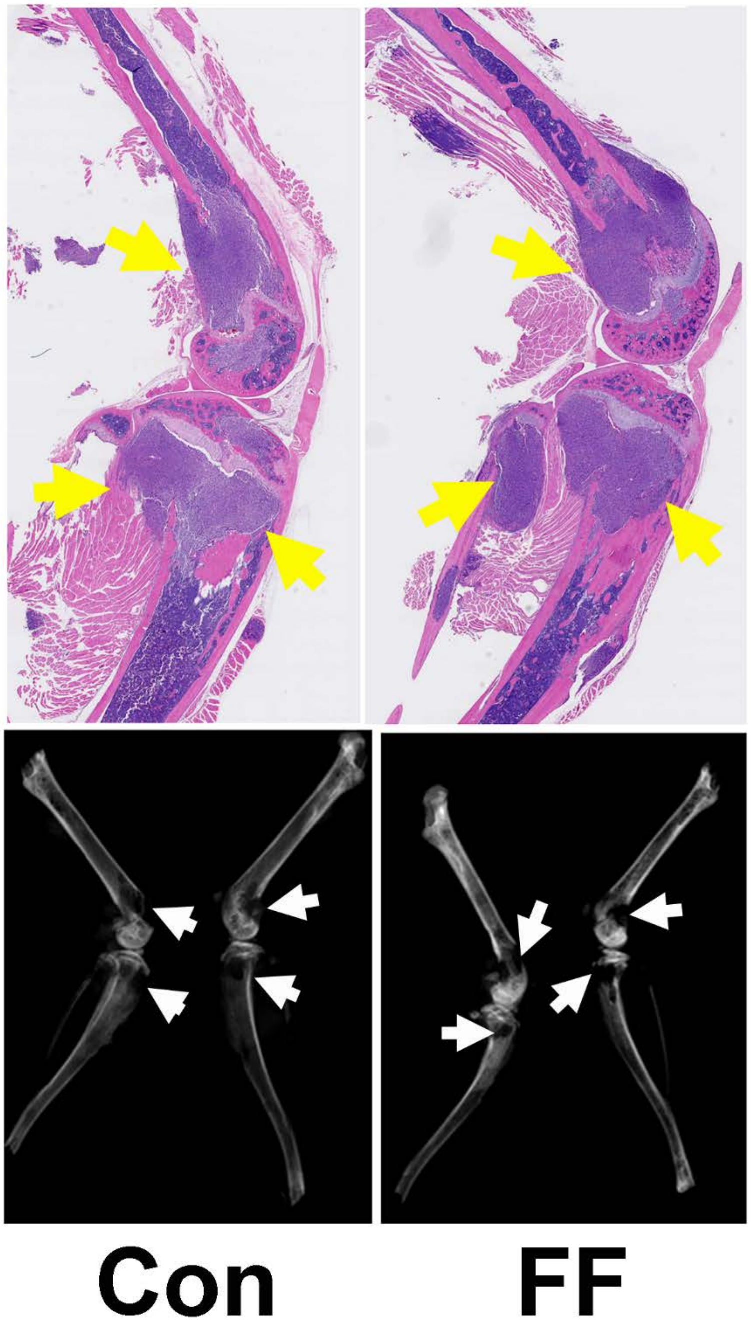

Fig S1: 2 months old FF and control mice were injected with Bo1 cell via left ventricular chamber. 12 days later, the tumor burden in bone was analyzed by HE histology and radiographs. Arrows indicate metastasis.

Figure S2

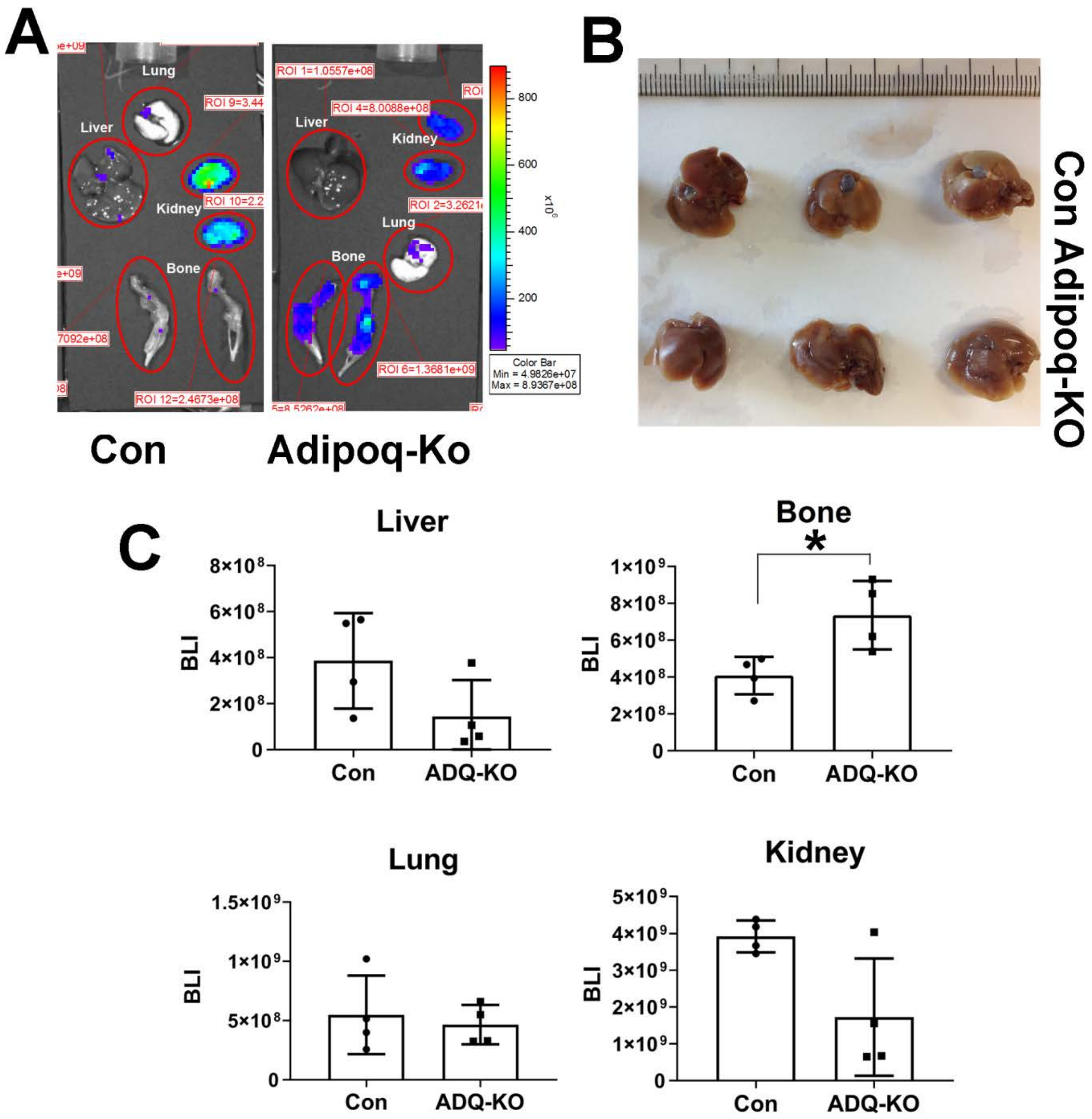

Fig S2: 2 month old adiponectin-/- (Adipoq-KO) and control (Con) mice were injected with Bo1 cell via left ventricular chamber.12 days later tumor burden was analyzed: A) *ex vivo* image of liver, bone, lung and kidney; B) Gross appearance of Con and Adipoq-KO liver; C) Quantification of tumor burden in liver, bone, lung and kidney of Con and Adipoq-KO mice. Data are presented as mean ± SD. n=4. *P<0.05, as determined by unpaired t test.

Figure S3

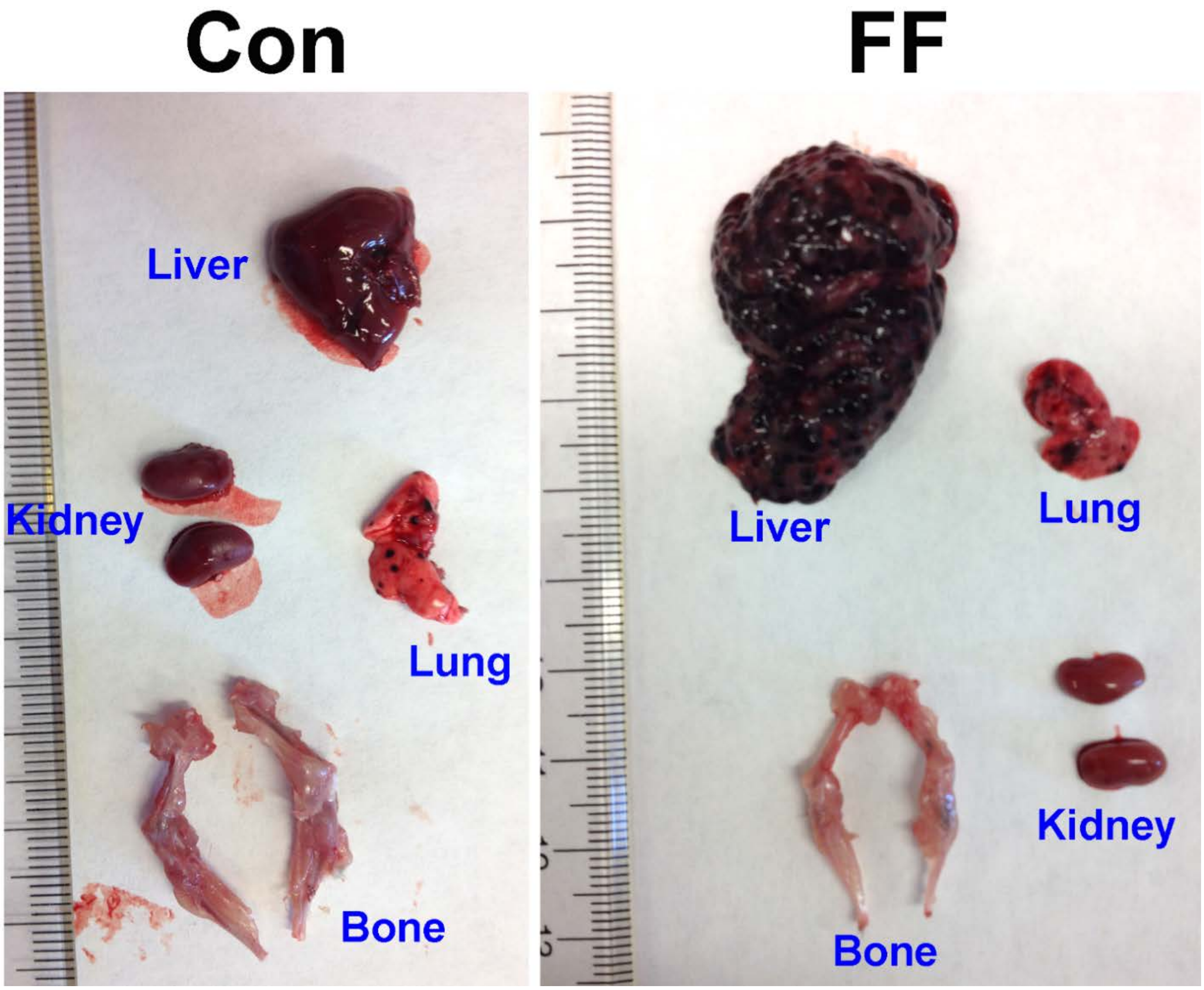

Fig S3: Gross appearance of liver, lung, kidney and bone of control (Con) and FF mice. 12 days after intracardiac injection of B16 melanoma cells.

Figure S4

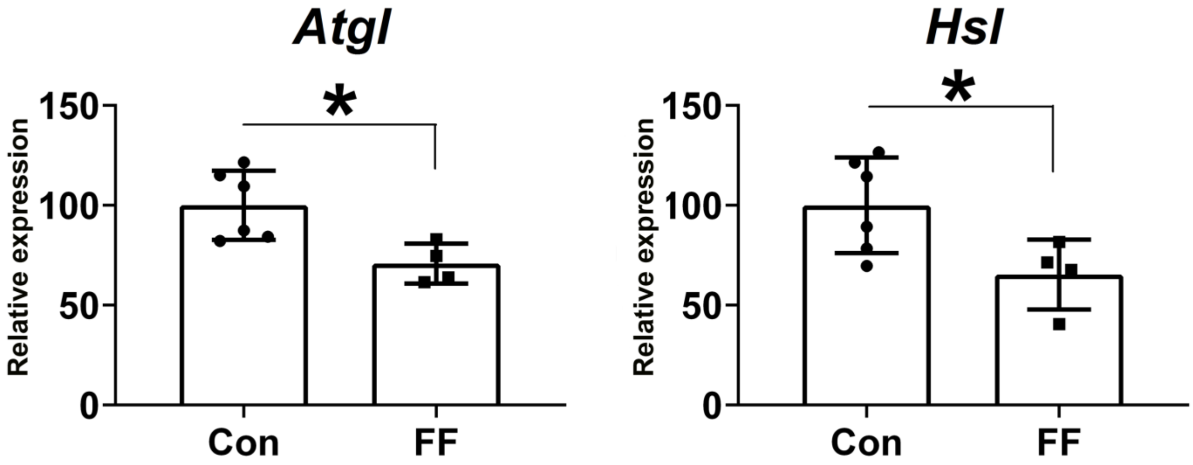

Fig S4: Expression of ATGL and HSL mRNA of control (Con) or FF liver explants when cultured in the absence of Bo1 cells. Data are presented as mean ± SD. n=4-6. * $p<0.05$ as determined by unpaired t test.

Figure S5

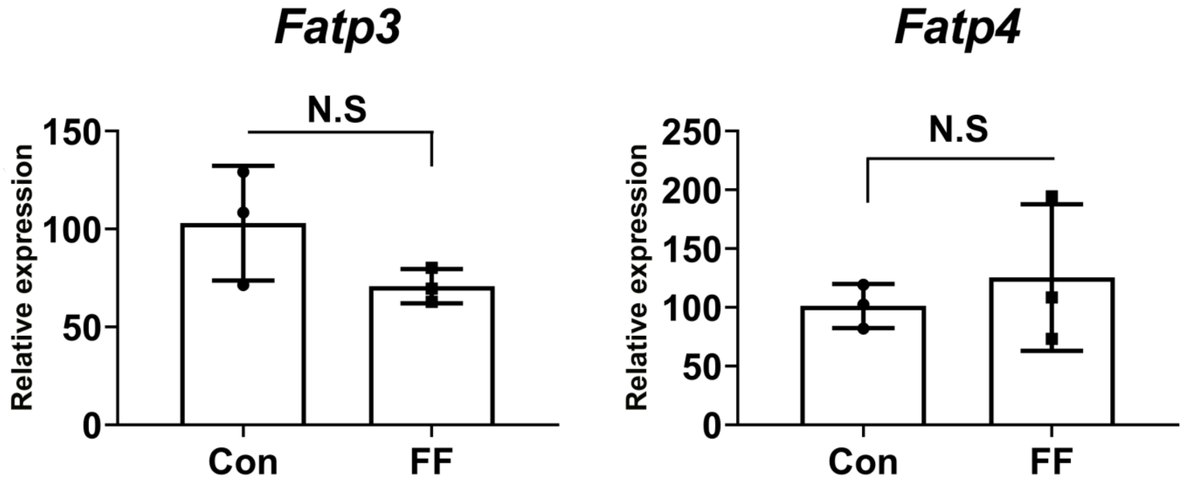

Fig S5: Expression of FATP3 and FATP4 mRNA in Bo1 cells exposed to control (Con) or steatotic (FF) liver conditioned medium. Data are presented as mean ± SD. n=3. P value was determined by unpaired t test.

Figure S6

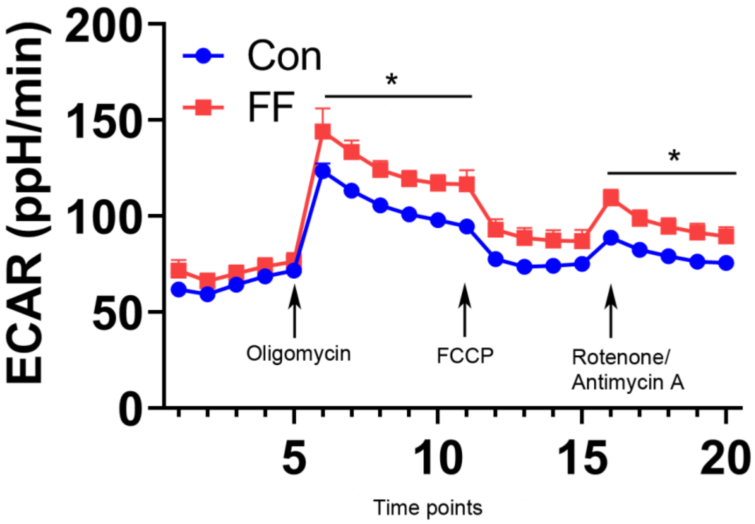

Fig S6: Extracellular acidification rate (ECAR) assay of Bo1 cells co-cultured with control (Con) or fatty (FF) liver. Data are presented as mean ± SD. n=9. * $p<0.05$ as determined by unpaired t test.

Figure S7

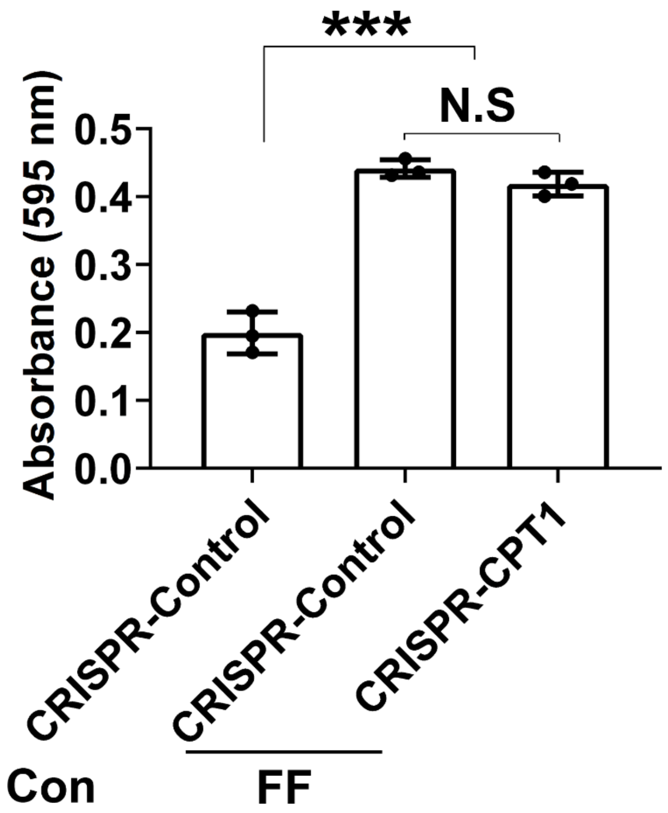

Fig S7: CRISPR control or CRISPR-CPT1 knockout Bo1 cells were cultured with control (Con) or FF liver explants in transwell system and migrating cells number quantified. Data are presented as mean ± SD. n=3. *** p<0.001 as determined by one way ANOVA test with analysis of variance with Holm-Sidak's multiple comparisons test.
